# Optimization of Salt Composition and Influence of β‐Lactoglobulin Nanostructures on the Physicochemical and Textural Properties of Minas Frescal Cheese

**DOI:** 10.1111/jtxs.70118

**Published:** 2026-10-11

**Authors:** Brunna D'Onofre Couto, Igor José Boggione Santos

**Affiliations:** ^1^ Department of Chemistry, Biotechnology and Bioprocess Engineering Federal University of São João del‐Rei Ouro Branco MG Brazil

**Keywords:** low sodium, Minas fresh cheese, nanostructures, quercetin, vitamin B_2_, β‐Lactoglobulin

## Abstract

Excessive sodium consumption is a primary driver of modern health adversities, prompting the dairy industry to seek reduction strategies that do not compromise product integrity. This study aimed to investigate the influence of β‐lactoglobulin (β‐lg) nanostructures, containing encapsulated quercetin and vitamin B2, on the functional and structural profile of low‐sodium Minas Frescal cheese. Using a central composite rotatable design (CCRD), the research evaluated the effects of three independent variables: sodium chloride (NaCl; 5–20 g L^−1^) concentration, potassium chloride (KCl; 5–20 g L^−1^) concentration, and β‐lg nanostructure concentration (5–10 mg kg^−1^). Among the evaluated responses, pH, titratable acidity, cohesiveness, springiness, hardness, and chewiness were not significantly affected by the incorporation of β‐lg nanostructures. In contrast, electrical conductivity ranged from 1.28 to 3.92 mS cm^−1^ and was significantly influenced by the salt composition, with KCl showing the greatest effect, while adhesiveness was primarily affected by NaCl concentration. The optimized formulation consisted of 2.5% total salt (50% NaCl and 50% KCl) supplemented with 7.5 mg kg^−1^ β‐lg nanostructures, maintaining the physicochemical and textural characteristics of conventional Minas Frescal cheese. These findings demonstrate that β‐lg nanostructures provide a stable delivery platform for bioactive compounds and enable the development of low‐sodium Minas Frescal cheese without compromising product quality.

## Graphical abstract

A central composite rotatable design was used to evaluate the effects of sodium chloride (NaCl), potassium chloride (KCl), and β‐lactoglobulin (β‐lg) nanostructure concentrations on the physicochemical and textural properties of Minas Frescal cheese. An optimized formulation containing 2.5% total salt (50% NaCl and 50% KCl) and 7.5 mg/kg β‐lg nanostructures was found to maintain the cheese's physicochemical and textural characteristics. β‐Lg nanostructures enabled the incorporation of quercetin and vitamin B2 without compromising product quality.

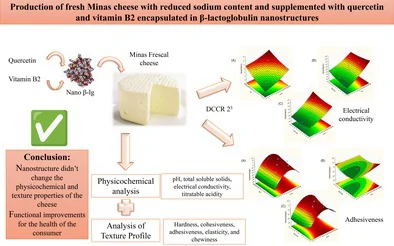

## 1 Introduction

In recent years, increasing consumer demand for healthier, high‐quality foods has driven the development of innovative products that combine improved nutritional value with desirable technological characteristics (Elsebaie et al. 2025). In this context, reducing sodium content has become a major research priority due to public health recommendations, as excessive salt intake is strongly associated with hypertension and cardiovascular diseases, the leading causes of mortality worldwide (Hunter et al. 2022).

To address this challenge, recent studies have explored strategies to reduce sodium while preserving product quality, including the incorporation of plant‐derived ingredients in bakery products (Elsebaie et al. 2025). In dairy products, for example, bioactive approaches such as the incorporation of postbiotics and zinc‐carbon dots have successfully extended the shelf life of fresh cheeses while maintaining their microbiological and sensory quality (Divsalar et al. 2025). These advances reflect the growing interest in developing healthier and multifunctional foods while maintaining their technological performance, particularly, in cheese, one of the processed foods with the highest sodium content (Ferroukhi et al. 2023).

Minas Frescal cheese relies on sodium for preservation, texture, and flavor. However, reducing sodium chloride (NaCl) is technically challenging because it may compromise these properties (Gomes et al. 2011; Felicio et al. 2016). The type and concentration of mineral salts like sodium, potassium, and calcium influence protein interactions, water retention, and the structural stability of food matrices, which directly affects texture and physicochemical properties (Ataollahi Eshkour et al. 2023; Yousefi et al. 2025). Among sodium reduction strategies, partially replacing NaCl with potassium chloride (KCl) has shown promising results. This method reduces sodium by approximately 40%–50% while preserving the quality of several cheese varieties when the substitution level is maintained between 25% and 50% (Gomes et al. 2011; Costa et al. 2018; Golin Bueno Costa et al. 2019). However, higher proportions of KCl may impair sensory acceptance due to bitterness, a metallic taste, and textural changes (Jahić et al. 2024). Therefore, optimizing the NaCl/KCl ratio is crucial to balancing sodium reduction without compromising the food's physicochemical and textural properties.

In this context, nanotechnology offers a promising frontier for the food sector, particularly, through the incorporation of bioactive molecules (El‐Sayed and El‐Sayed 2021) and the enhancement of food architecture, including viscosity, shelf life, and overall sensory quality (Livney 2010).

The efficacy of nanoencapsulation depends on the molecular interactions between the carrier and guest molecules. For this study, β‐lactoglobulin (β‐lg), as a globular whey protein, was chosen as the carrier because of its amphiphilic nature. This property enables the encapsulation and stabilization of both hydrophobic and hydrophilic bioactive compounds (Huang et al. 2010; Durán and Marcato 2013; Milea et al. 2020). Recent studies have further demonstrated the potential of nanostructured systems for the stabilization and delivery of hydrophobic flavonoids, such as kaempferol, highlighting the versatility of nanoscale carriers for bioactive compounds (Zhang et al. 2026).

Furthermore, environmental conditions, particularly, pH and ionic composition, strongly influence the structural and functional behavior of β‐lg by affecting protein conformation and intermolecular interactions. Recent studies have demonstrated that calcium ions and hydrocolloids can significantly alter the rheological and structural properties of β‐lg systems. Similar effects of calcium incorporation on the physicochemical behavior of cross‐linked macromolecular networks have also been reported in calcium‐doped hydrogel systems, highlighting the broader role of ionic interactions in modulating the properties of structured polymeric and biopolymeric matrices (Wang et al. 2026). This underscores the importance of evaluating β‐lg nanostructures under various ionic conditions, such as those resulting from replacing NaCl with KCl, to ensure compatibility with the cheese matrix (Rafe and Razavi 2013; Rafe et al. 2022).

Specifically, quercetin, a flavonoid with potent antioxidant and antimicrobial properties (Caruana et al. 2022), interacts with the internal hydrophobic cavity of β‐lg via van der Waals forces. Conversely, riboflavin (Vitamin B2), which is essential for human metabolism but cannot be synthesized by the body (Mack and Grill 2006), associates with the protein's surface through hydrogen bonding and electrostatic interactions. These distinct binding modes stabilize the bioactives without disrupting the supramolecular organization of the protein carrier.

Although β‐lg nanostructures are not intended to replace sodium chloride's technological functions, they may interact with cheese's protein network and should therefore be evaluated under different ionic conditions created by replacing NaCl with KCl. Changes in ionic strength and cation composition can affect protein–protein interactions, water distribution, and matrix organization, which can impact the incorporation and stability of protein‐based nanostructures (Jahić et al. 2024). Thus, evaluating the compatibility of β‐lg nanostructures with reduced‐sodium cheese formulations is essential for developing functional dairy products enriched with bioactive compounds that maintain desirable physicochemical and textural characteristics.

The objective of this work was to investigate the influence of β‐lg nanostructures, both as empty carriers and loaded with nutraceuticals, on the physicochemical and textural properties of Minas Frescal cheese. By focusing on the role of these protein nanostructures as functional nanocarriers, this study aims to elucidate how their presence modulates the complex dairy matrix.

## 2 Materials and Methods

### 2.1 Material

β‐lg isolated from bovine whey was generously supplied by Davisco Food International in Minnesota, USA. The protein preparation contained 97.8% protein on a dry basis (w/w), with β‐lg accounting for 93.6% of the total protein content. Sodium chloride (NaCl) and potassium chloride (KCl) used were of analytical grade from Exodus Scientific (São Paulo, Brazil). Commercial pasteurized whole cow's milk was used for cheese manufacture, and rennet (chymosin enzyme) brand HA‐LA was used in the preparation of Minas Frescal cheese. The distilled water was purified by Hidrotek reverse osmosis (Santa Iria de Azóia, Portugal). The other reagents were of analytical grade without further purifications.

### 2.2 Methods

#### 2.2.1 Nanoprotein Synthesis and Encapsulation of Bioactives

The β‐lg protein was dissolved at a concentration of 2 mg mL^−1^ in a 50 mmol L^−1^ sodium chloride solution at pH 7.10 ± 0.01. The resulting solution was subjected to thermal treatment in an ultrathermostatic water bath at 100°C for 60 min to promote the formation of β‐lg nanostructures. The experimental details were described in (D'Onofre Couto et al. 2021).

The encapsulation of bioactives was also described in (D'Onofre Couto et al. 2021). Quercetin solutions were prepared at a concentration of 0.993 mmol L^−1^, and Vitamin B_2_ solutions were prepared at a concentration of 0.04 mmol L^−1^. The bioactive compounds were individually mixed with the β‐lg nanostructure dispersion, manually homogenized for 5 min, and centrifuged (20,579*g*, 20°C, 30 min). The resulting pellet was dried at 50°C for 48 h. Encapsulation efficiency (EE) and loading capacity (LC) were determined by UV–Vis spectrophotometry using the corresponding absorption wavelengths and calculated as previously described.

#### 2.2.2 Experimental Design

The optimization of the cheese production was carried out through the experimental design of a (CCRD), 23 fractions, with 8 experiments at 2 levels, for a total of 17 trials, with 6 experiments at the axial points and 3 replications at the central point. The independent variables were the concentrations of β‐lg nanostructures, NaCl and KCl, as shown in Table 1. The matrix of bounds and levels for each of these variables is shown in Supporting Information, Table A.1. The response variables were pH, conductivity (mS cm^−1^), titratable acidity (% lactic acid), cohesiveness, adhesiveness (g s), elasticity, hardness (N), chewiness (N). These data were processed using statistical software. Control cheeses, that is, without nanostructure (Supporting Information, Table A.2.), maintaining the concentrations of KCl and NaCl from the experimental design and evaluating the same parameters. These control formulations were included as a reference basis to compare the potential modifications in the physicochemical and textural properties of the cheese resulting from the incorporation of β‐lg nanostructures.

**TABLE 1:** Experimental design of rotatable central composite design (DCCR) 23, with 8 experiments on 2 levels, for a total of 17 trials, with 6 experiments at the axial points and 3 replicates at the central point, having as independent variables the concentrations of sodium chloride (NaCl), potassium chloride (KCl), and β‐lg nanostructures.

Test	Encoded variables			Decoded variables		
	NaCl (g L^−1^)	KCl (g L^−1^)	Nano β‐lg (mg Kg^−1^)	NaCl (g L^−1^)	KCl (g L^−1^)	Nano β‐lg (mg Kg^−1^)
1	−1	−1	−1	5	20	5
2	−1	1	1	5	5	10
3	1	−1	1	20	20	10
4	1	1	−1	20	5	5
5	0	0	0	12.5	12.5	7.5
6	−1	−1	1	5	20	10
7	−1	1	−1	5	5	5
8	1	−1	−1	20	20	5
9	1	1	1	20	5	10
10	0	0	0	12.5	12.5	7.5
11	−1.67332	0	0	0	12.5	7.5
12	1.67332	0	0	25	12.5	7.5
13	0	−1.67332	0	12.5	25	7.5
14	0	1.67332	0	12.5	0	7.5
15	0	0	−1.67332	12.5	12.5	3.32
16	0	0	1.67332	12.5	12.5	11.68
17	0	0	0	12.5	12.5	7.5

#### 2.2.3 Minas Frescal Cheese Production

Minas Frescal cheese was produced according to the flow chart in Supporting Information, Figure A.1. The production process followed the methodology described in (Moreira et al. 2021) with adaptations. Yeast and lactic acid were not added to analyze the interference of the addition of nanostructure with and without bioactives and KCl on the production process of Minas Frescal cheese.

The addition of salt, calcium chloride (CaCl_2_), and the nanostructure was carried out in commercial pasteurized whole cow's milk before the heating step. Each cheese was made with 500 mL of milk. The added masses of salt and nanostructure were calculated according to the DCCR; rennet was added according to the manufacturer's instructions Ha‐La and calcium chloride (CaCl_2_) at 0.02% (m v^−1^) on the volume of milk. To produce bioactive cheeses, first, each bioactive was encapsulated in the nanostructure, and the resulting pellet was resuspended and applied in the production process. These bioactive cheeses were produced in the best condition found through DCCR planning.

#### 2.2.4 Physicochemical Analysis of Cheese With and Without β‐lg Nanostructure

For the physical–chemical analyses, the tests were carried out in triplicate and using the methodology recommended by the Association of Official Analytical Chemists (AOAC).

##### 2.2.4.1 pH

The cheeses were submitted to pH determination analysis using a Digimed pHmeter (Brazil). For this, 5.0 g of cheese was added to 50.0 mL of distilled water and crushed for homogenization and further analysis.

##### 2.2.4.2 Electrical Conductivity

The electrical conductivity (mS cm^−1^) was evaluated in a conductivity meter (Tecnopon MCA 150). For this, 5.0 g of cheese was added to 50.0 mL of distilled water and crushed for homogenization and further analysis.

##### 2.2.4.3 Titratable Acidity

Acidity was determined to identify the lactic acid content and calculated by Equation (1).

(1) $$\%\text{Titratable acidity}=\frac{V\times f\times 0.9\;}{m}$$

where *V* is the volume of NaOH spent in the titration; *f* is the NaOH factor solution; 0.9 is the lactic acid factor, and *m* is the cheese mass.

#### 2.2.5 Analysis of Cheese Texture Profile

##### 2.2.5.1 Instrumental Texture Profile Analysis (TPA)

Instrumental TPA was performed according to the methods described by Queiroga et al. (2013) and Hesarinejad et al. (2021), with minor modifications. The cheese samples were approximately 5.8 cm in diameter, 2.5 cm in height, and analyzed on the third day after production. The samples were refrigerated at 4°C until analysis. The TA.XT Plus Texture Analyzer (Stable Micro Systems Ltd., Godalming, UK) was used at 25°C. The samples underwent a double compression test at 50% of their original height using a cylindrical probe (10 cm diameter × 10 mm height). The pretest, test, and posttest speeds were set at 2.0 mm/s, with a 5‐s interval between the two compression cycles. The following properties were calculated from force‐time curves using the equipment's software: hardness (maximum force required for compression), cohesiveness (ratio of work performed during the second compression to that of the first), adhesiveness (negative work required to overcome attractive forces between the sample and the probe), springiness (degree of recovery of the sample's original height after the first compression), and chewiness (product of hardness, cohesiveness, and springiness) (Hesarinejad et al. 2021). Each cheese formulation was analyzed once, as the study involved specific trials based on a central composite rotatable design (CCRD).

#### 2.2.6 Statistical Analysis

The results were statistically analyzed using the statistical software. Analysis of variance (ANOVA), with a 95% confidence level, was used to determine how the independent variables of the DCCR affected the values of the response variables. For the statistical analysis of the comparisons of the planning cheeses with the control cheeses, the Tukey test was used at a 5% significance level, which is a method for paired comparison of results.

## 3 Results and Discussion

### 3.1 Physicochemical Analysis of Cheese With and Without β‐Lg Nanostructure

#### 3.1.1 pH, Total Soluble Solids, and Titratable Acidity

Table 2 shows the pH and titratable acidity of cheeses produced by DCCR with nanostructure and the same parameters for control cheeses.

**TABLE 2:** Physicochemical analysis of cheeses with different concentrations of β‐lg nanostructures, NaCl, and KCl according to DCCR depending on the response variables pH, titratable acidity (%), electrical conductivity (mS.cm^−1^).

	pH	Titratable acidity (%)	Electrical conductivity (mS cm^−1^)
*DCCR test*			
1	6.58 ± 0.02^b,c,d^	0.60 ± 0.02^a^	2.87 ± 0.09^g,h^
2	6.65 ± 0.06^b,c,d,e,f^	0.58 ± 0.00^a^	1.31 ± 0.04^a,b^
3	6.61 ± 0.04^b,c,d^	0.58 ± 0.07^a^	3.84 ± 0.04^k^
4	6.64 ± 0.03^b,c,d,e^	0.66 ± 0.04^a,b^	2.28 ± 0.02^e^
5	6.87 ± 0.03^f,g,h^	0.60 ± 0.02^a^	2.43 ± 0.07^e,f^
6	6.30 ± 0.08^a^	0.92 ± 0.02^d^	2.91 ± 0.07^g,h^
7	6.44 ± 0.04^a,b^	0.83 ± 0.03^c,d^	1.28 ± 0.08^a^
8	6.59 ± 0.05^b,c,d^	0.70 ± 0.03^a,b^	3.92 ± 0.07^k^
9	6.58 ± 0.05^b,c^	0.79 ± 0.03^b,c^	2.82 ± 0.05^g,h^
10	6.89 ± 0.03^g,h^	0.60 ± 0.02^a^	2.41 ± 0.09^e,f^
11	6.84 ± 0.03^g,h^	0.70 ± 0.02^a,b,c^	1.68 ± 0.07^c,d^
12	6.87 ± 0.04^f,g,h^	0.68 ± 0.01^a,b^	3.34 ± 0.07^j^
13	6.87 ± 0.02^g,h^	0.70 ± 0.03ª^,b,c^	3.28 ± 0.03^i,j^
14	6.87 ± 0.00^g,h^	0.65 ± 0.02^a^	3.28 ± 0.08^d^
15	6.95 ± 0.03^h^	0.60 ± 0.01^a^	2.40 ± 0.09^e,f^
16	6.80 ± 0.05^d,e,f,g,h^	0.66 ± 0.02^a,b^	2.40 ± 0.09^e,f^
17	6.92 ± 0.03^h^	0.64 ± 0.02^a^	2.40 ± 0.08^e,f^
*Control test*			
1	6.80 ± 0.08 ^d,e.f.g,h^	1.12 ± 0.03^e,f^	2.98 ± 0.04^h,i^
2	6.69 ± 0.28^c,d,e,f,g^	1.09 ± 0.05^e,f^	1.39 ± 0.12^a,b,c^
3	6.85 ± 0.02^e,f,g,h^	1.06 ± 0.03^e^	4.43 ± 0.06^l^
4	6.95 ± 0.06^h^	1.13 ± 0.03^e,f^	2.68 ± 0.32^f,g,h^
5	6.87 ± 0.02^f,g,h^	1.19 ± 0.02^f^	2.61 ± 0.05^f,g^
6	6.95 ± 0.05^h^	1.17 ± 0.08 ^e,f^	1.61 ± 0.00^b,c,d^
7	6.86 ± 0.04^f,g,h^	1.09 ± 0.00 ^e,f^	4.14 ± 0.16^k,l^
8	6.84 ± 0.04^e,f,g,h^	1.09 ± 0.08 ^e,f^	4.41 ± 0.02^l^
9	6.78 ± 0.06^c,d,e,f,g,h^	1.17 ± 0.12 ^e,f^	1.76 ± 0.08^d^

*Note:* Different superscripts (a, b, c, d, e, f, g, h, i, j, k) differ significantly at *p* ≤ 0.05 according to the Tukey test for each response variable also comparing with controls.

The Tukey test identified statistically significant differences in pH between some control cheeses (without β‐lg nanostructures) and the CCRD formulations. However, the overall pH variation was small, ranging from 6.30 to 6.95. These differences may be due to variations in the activity of acid‐producing bacteria during cheese production because microbial fermentation of lactose can lower pH by producing organic acids (Moreira et al. 2020). The pH values obtained in the present study are consistent with those reported for Minas Frescal cheese by Silva et al. (2020), who observed values between 6.2 and 6.8 during production. However, these values are higher than those reported by Moreira et al. (2021) for reduced‐sodium artisanal Minas Frescal cheese (5.1–5.5) (Moreira et al. 2021; Silva et al. 2020). Additionally, the Pareto chart (Supporting Information, Figure A.2.) revealed that pH was not a significant response variable in the CCRD model. This indicates that neither the β‐lg nanostructures nor the NaCl/KCl combinations significantly affected this Minas Frescal cheese physicochemical property.

Regarding titratable acidity, only cheeses six and seven differed statistically from the remaining formulations, although they did not differ from each other according to Tukey's test. Furthermore, the Pareto chart (Supporting Information, Figure A.3.) indicated that titratable acidity was not a significant response variable in the CCRD model. The cheeses exhibited low lactic acid contents, ranging from 0.58% to 0.92%, which is consistent with previous reports for Minas Frescal cheese (Silva et al. 2020). Titratable acidity is mainly associated with lactose fermentation by lactic acid bacteria naturally present in milk or introduced during cheese manufacture (Matera et al. 2018). In the present study, no starter cultures were added, and the relatively high concentrations of NaCl and KCl likely limited microbial growth by reducing water activity (Yousefi et al. 2025), thereby restricting lactic acid production (Oliveira et al. 2017). The statistical difference occurs between the design and control cheeses, which have higher lactic acid levels ranging from 1.09% to 1.19%. The lower titratable acidity observed in cheeses containing β‐lg nanostructures may be related to changes in the cheese matrix's microstructure and buffering properties, rather than differences in acid production. Nano‐β‐lg interacts with casein through hydrophobic interactions and disulfide bonds, which alter the protein network (Zhang et al. 2024). Furthermore, incorporating β‐lg nanostructures increases the protein content of the aqueous phase and introduces additional ionizable amino acid residues that can modify the acid–base balance of the system. These interactions can affect the amount of alkali needed for titration without altering the pH, which remained consistent throughout the treatments (Fox et al. 2017).

#### 3.1.2 Electrical Conductivity

Electrical conductivity differed significantly among the CCRD formulations according to Tukey's test (Table 2). Cheeses containing higher salt concentrations (Tests 3, 8, 12, and 13) exhibited the highest conductivity values (3.34–3.92 mS cm^−1^), whereas formulations with lower salt concentrations (Tests 2 and 7) showed the lowest values (1.28–1.31 mS cm^−1^). The Pareto chart (Supporting Information, Figure A.4.) confirmed that both NaCl and KCl concentrations significantly influenced electrical conductivity, with KCl exerting the greatest effect. This finding is consistent with the higher molar conductivity of KCl (14.986 S cm^2^ mol^−1^) compared with NaCl (12.645 S cm^2^ mol^−1^) at infinite dilution (Bald et al. 2013).

In addition to differences in molar conductivity, the higher conductivity observed in KCl, containing formulations may also reflect the weaker hydration tendency of K^+^ relative to Na^+^. According to the Hofmeister series, Na^+^ ions bind water more strongly and compete more effectively with proteins for available water molecules, whereas K^+^ ions remain comparatively more mobile in the aqueous phase of the cheese matrix (Carr et al. 2002; El‐Bakry et al. 2011). This ion‐specific behavior is consistent with the higher electrical conductivity observed in formulations with greater KCl content.

According to the ANOVA (Supporting Information, Table A.3.), the highest *F* values were for KCl, and therefore it is the main influence in obtaining the electrical conductivity value. The *p*‐values were small, highlighting the reliability of the model. The value of *F*_calculated_ was obtained equal to 156.04 and *F*_tabulated_ was equal to 3.79, so the data is statistically significant.

The response surfaces (Figure 1 and Supporting Information, Figures A.5.–A.7.) showed that electrical conductivity increased with increasing NaCl and KCl concentrations. Since the objective of this study was to reduce sodium while maintaining the technological functions of salts in cheese, formulations with intermediate salt concentrations represented the most suitable region for optimization.

**FIGURE 1:**
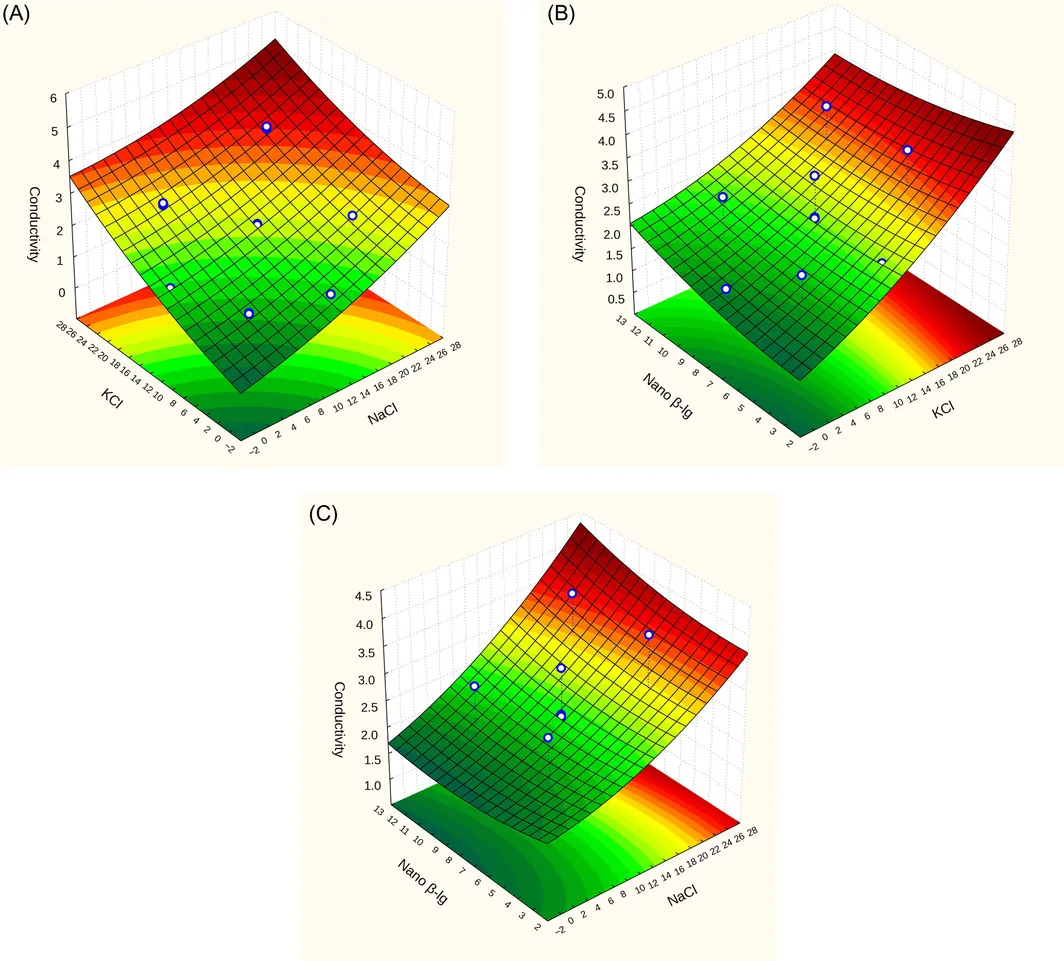
3D response surface of the three‐factor DCCR with the dependent variable electrical conductivity as a function of the coded variables (A) concentration of NaCl and KCl; (B) concentration of KCl and β‐lg nanostructures; (C) concentration of NaCl and β‐lg nanostructures.

Significant differences were also observed between cheeses containing β‐lg nanostructures and their respective control formulations. For example, controls C3, C7, and C8 exhibited higher electrical conductivity than their corresponding cheeses produced with the same salt concentrations (Tests 3, 8, 12, and 13). This result suggests that the incorporation of β‐lg nanostructures reduced ionic mobility within the cheese matrix, possibly by modifying the protein network and restricting ion diffusion, thereby decreasing electrical conductivity despite equivalent salt contents.

### 3.2 Analysis of Cheese Texture Profile

#### 3.2.1 Instrumental TPA

TPA simulates the compressions exerted on a piece of cheese when it is chewed, generating a force‐per‐time profile that determines the mechanical properties (Fox et al. 2017) and the results are shown in Table 3.

**TABLE 3:** Instrumental texture analysis of cheeses with different concentrations of β‐lg nanostructures, NaCl and KCl, following DCCR and Control Tests, depending on the response variables cohesiveness, adhesiveness (g.s), elasticity, chewability (N), hardness.

	Cohesiveness	Adhesiveness (g s)	Elasticity	Chewiness (N)	Hardness (N)
*DCCR test*					
1	0.6934	−3.7875	0.7779	46.2424	85.7281
2	0.7300	−5.5016	0.8258	43.4630	72.0925
3	0.7781	−0.7022	0.8283	41.7737	64.8172
4	0.7474	−3.1866	0.7950	39.6590	66.7449
5	0.7916	−2.0746	0.8038	36.0204	76.1360
6	0.6341	−10.0612	0.7711	33.7630	69.0546
7	0.6581	−4.3749	0.7720	43.7702	86.1450
8	0.7126	−1.6851	0.7763	53.2121	96.1853
9	0.7468	−1.9391	0.7863	55.9334	95.2504
10	0.7259	−2.1943	0.8019	38.8408	73.8437
11	0.7203	−37.2442	0.8325	55.6475	92.7955
12	0.7873	−3.1688	0.8465	50.2940	75.4590
13	0.7648	−0.1119	0.8094	43.8309	70.8047
14	0.6155	−9.6918	0.8091	53.7861	98.0011
15	0.7426	−2.7544	0.7920	52.8128	89.7937
16	0.7718	−1.5678	0.7631	51.3175	87.1355
17	0.8125	−2.2523	0.8108	44.7319	66.8180
*Control test*					
1	0.7377	−2.2837	0.8244	49.5975	81.5498
2	0.4875	−7.9372	0.7868	35.3535	92.1619
3	0.4701	−3.7003	0.8215	35.4188	91.7287
4	0.5214	−1.5479	0.7928	29.9542	72.4634
5	0.5356	−15.1146	0.7662	34.0250	84.8416
6	0.6077	−23.8696	0.8208	45.0100	90.2445
7	0.5745	−4.0059	0.7448	35.9423	78.5201
8	0.6191	−4.4257	0.7968	54.4398	114.7814
9	0.4664	−6.8780	0.8728	62.6258	153.845

The Pareto chart (Supporting Information, Figure A.8.) revealed that cohesiveness was not a statistically significant response variable in the CCRD model. Despite variations in salt concentrations and β‐lg nanostructure levels, cohesiveness remained relatively constant, ranging from 0.6155 to 0.8125. This indicates that the structural integrity of the cheese matrix was preserved. These values are consistent with those reported for Coalho cheese produced with a 1:1 NaCl/KCl ratio (Costa et al. 2018) and for Minas Frescal cheese made from sheep's milk (Borges et al. 2019).

Compared with the corresponding control cheeses, formulations containing β‐lg nanostructures generally exhibited higher cohesiveness at equivalent salt concentrations. For instance, Assays 2 and 7 had an average cohesiveness of 0.6941, while control C2 had a value of 0.4875. A similar trend was observed for Assays 3 and 8 relative to control C3. These results suggest that incorporating β‐lg nanostructures contributes to preserving the continuity of the protein matrix by reinforcing intermolecular interactions and reducing structural disruption.

Similarly, elasticity was not significantly affected by the experimental variables (Supporting Information, Figure A.9.), with values ranging from 0.7631 to 0.8465. The elasticity and cohesiveness parameters are related, as the more elastic the cheese, the greater its resistance to breakage, and therefore its cohesiveness. Test 12, which has the highest concentration of NaCl, was the test with the highest elasticity (0.8465), and it was expected to be the lowest value, since high levels of NaCl can cause an increase in the expansion of the protein network, as, more water is retained between the salt molecules, reducing the elasticity of the cheese and making it more brittle. When water binds to casein, it acts as a plasticizer, that is, a lubricant of the protein network, making the protein matrix less elastic, more fragile, and less firm (Lucey et al. 2003). Probably, NaCl is not free to cause this expansion of the protein network of the cheese, but is encapsulated in the β‐lg nanostructure, which interferes with the mass transfer processes, which can reduce the water activity and, consequently, the moisture content, maintaining the elasticity values (Zhang et al. 2024).

It should be noted that for all the DCCR tests, high values of hardness and, consequently, chewability were obtained, and both were not a statistically significant response for the DCCR applied (Supporting Information, Figures A.10 and A.11). The hardness values obtained are within the range reported for similar fresh cheeses, although considerable variability exists among studies due to differences in milk composition, moisture and fat contents, salt concentration, starter cultures, and coagulants (Queiroga et al. 2013; Costa et al. 2018). These results can be justified by the fact that Minas Frescal cheese is very heterogeneous, with differences in the production process and operational parameters (Oliveira et al. 2017) Comparison with the control cheeses also revealed similar textural behavior. However, assay 13 exhibited lower hardness than control C8 despite having the same NaCl/KCl concentration, suggesting that the incorporation of β‐lg nanostructures may have contributed to a slight attenuation of cheese firmness without compromising its overall textural characteristics.

The adhesiveness response variable relates to the force required to remove the cheese from the palate while chewing occurs (Fox et al. 2017). For this response, there was statistical interference in the DCCR (Supporting Information, Figure A.12).

NaCl is the variable with the greatest effect on adhesion. This behavior can be explained by the influence of sodium on the mineral equilibrium of the cheese matrix. The addition of NaCl promotes the partial solubilization of colloidal calcium phosphate and the exchange of Na^+^ with Ca^2+^ ions associated with the casein network, modifying protein–protein interactions within the cheese matrix (Felicio et al. 2016)(Nagyová et al. 2014). Therefore, its application in the production process favors the peptization of the protein chain, the dispersion and hydration of proteins, in addition to the emulsification and stabilization of fat (Hoffmann et al. 2012), which are factors that directly affect the texture of the product.

This behavior can also be interpreted in light of the differential hydration tendencies of Na^+^ and K^+^. Because Na^+^ competes more strongly with casein for water molecules, it promotes greater solubilization of colloidal calcium phosphate and, consequently, higher casein hydration, resulting in a more cohesive and adhesive protein network. K^+^, being less effective at displacing water from the protein surface, produces a comparatively weaker effect on calcium equilibrium and casein hydration (Carr et al. 2002; El‐Bakry et al. 2011). This ion‐specific mechanism helps explain why NaCl, rather than KCl, was the dominant factor influencing adhesiveness in the CCRD model.

The ANOVA for the adhesiveness (Supporting Information, Table A.4.) shows the highest values of *F* were for NaCl and, therefore, it is the main factor influencing the adhesion, confirming the Pareto chart. The *p*‐values were small, highlighting the reliability of the model. The value of *F*_calculated_ was obtained equal to 14.51 and *F*_tabulated_ equal to 2.66, as *F*_calculated_ > *F*_tabulated_, it was possible to conclude that the data are statistically significant. From a technological perspective, formulations with intermediate adhesiveness are desirable, as this property is directly related to the characteristic texture of Minas Frescal cheese, which should present a moist, soft, homogeneous, and white matrix (Oliveira et al. 2017).

The response surface plots (Figure 2a–c; Supporting Information, Figures A.13–A.15) show that intermediate adhesiveness values were achieved at intermediate NaCl and KCl concentrations, regardless of the β‐lg nanostructure level. In particular, the yellow region of the response surfaces represents the optimal operating condition, combining moderate salt and nanostructure concentrations while maintaining the adhesiveness expected for Minas Frescal cheese.

**FIGURE 2:**
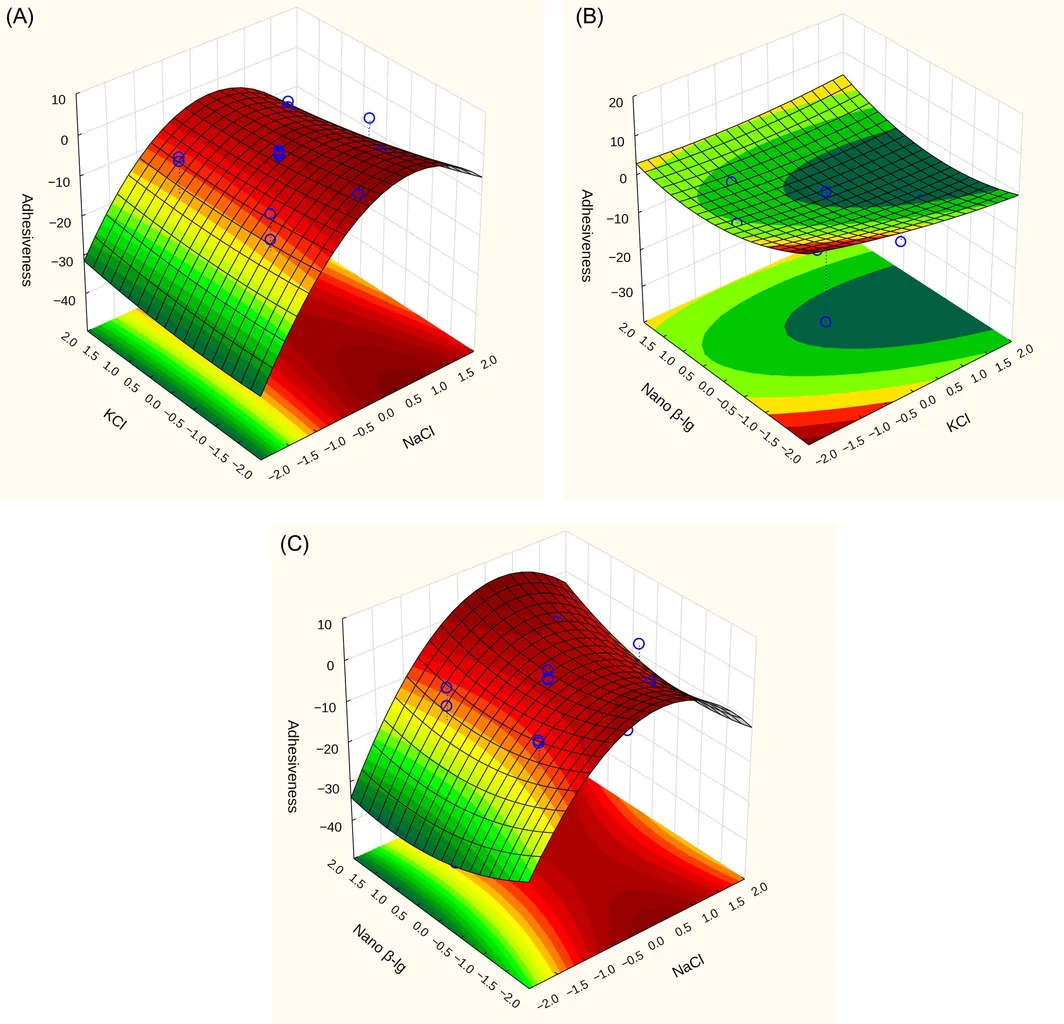
3D response surface of the three factor DCCR with the dependent variable adhesiveness as a function of the coded variables (A) concentration of NaCl and KCl; (B) concentration of KCl and β‐lg nanostructures; (C) concentration of NaCl and β‐lg nanostructures.

Nevertheless, formulation 11, in which NaCl was completely replaced by KCl, exhibited the highest adhesiveness, indicating a detrimental effect on cheese texture. This result is consistent with the findings of Alvarenga et al. (2008), who reported that NaCl replacement levels above 75% increased cheese firmness. This behavior has been attributed to changes in the mineral equilibrium of the cheese matrix, particularly, the interactions between sodium, calcium, and casein, which can reduce proteolysis and enhance protein dehydration, ultimately affecting textural properties (Alvarenga et al. 2008)(Ayyash et al. 2011).

Cheeses containing β‐lg nanostructures consistently exhibited lower adhesiveness than their respective controls at the same salt concentrations of KCl. This finding suggests that the incorporation of β‐lg nanostructures contributed to stabilizing the protein network and improving water distribution within the cheese matrix, thereby attenuating the increase in adhesiveness associated with high KCl levels. These results reinforce the technological potential of β‐lg nanostructures to preserve desirable textural characteristics while enabling sodium reduction in Minas Frescal cheese.

The central point of the CCRD was selected as the optimal formulation because it provided the best balance between the statistically significant response variables, electrical conductivity and adhesiveness. Although formulations two and seven contained the lowest salt concentration (1%), previous studies have shown that excessive sodium reduction may negatively affect consumer acceptance (Costa et al. 2018). Gomes et al. (2011) reported a 51.8% reduction in sodium content by replacing 25% of NaCl with KCl in Minas Frescal cheese, while maintaining satisfactory sensory acceptance. In the present study, the optimized formulation, containing 2.5% total salt (50% NaCl and 50% KCl) and 7.5 mg kg^−1^ of the nanostructure, preserved the physicochemical and textural characteristics of the cheese, demonstrating that partial replacement of NaCl with KCl, combined with the incorporation of β‐lg nanostructures, is a promising technological strategy for sodium reduction without compromising product quality.

In general, the maintenance of textural parameters in cheeses containing β‐lg nanostructures indicates that the nanoscale assemblies did not disrupt the casein network responsible for the structural organization of the cheese matrix. Instead, these nanostructures may act as colloidal fillers within the protein matrix, interacting weakly with casein micelles through electrostatic and hydrophobic interactions (Sadiq et al. 2021; Wang and Luo 2023). Such interactions can contribute to the stabilization of the protein network by promoting water retention and modulating ionic mobility within the matrix (Aguilar‐Pérez et al. 2023; Rathod et al. 2025).

### 3.3 Physicochemical and Texture Analysis of Minas Frescal Cheese With Bioactives Encapsulated in β‐Lg Nanostructure

Table 4 summarizes the physicochemical and textural properties of Minas Frescal cheeses containing β‐lg nanostructures loaded with either quercetin or vitamin B2, as well as the control formulation. There were no statistically significant differences in pH among the treatments, with values ranging from 6.45 to 6.68. Similarly, according to Tukey's test, titratable acidity did not differ significantly, indicating that the incorporation of bioactive‐loaded nanostructures did not affect the acid–base balance of the cheese matrix. The consistently low acidity values suggest that the manufacturing process effectively limited microbial fermentation and minimized lactic acid production during storage (Carvalho et al. 2007).

**TABLE 4:** Physicochemical analysis and instrumental texture analysis of quercetin nanoencapsulated cheese; vitamin B_2_ nanoencapsulated cheese and nonbioactive control as a function of response variables pH, titratable acidity (%), electrical conductivity (mS cm^−1^), cohesiveness, adhesiveness (g s), elasticity, chewability (N), hardness (N).

Test	pH	Titratable acidity (%)	Electrical conductivity (mS cm^−1^)
Quercetin	6.45 ± 0.13^a^	0.61 ± 0.05ª	2.43 ± 0.05^a^
Vitamin B_2_	6.63 ± 0.14^a,b^	0.62 ± 0.01^a^	2.40 ± 0.05^a^
Control	6.89 ± 0.03^b^	0.62 ± 0.02^a^	2.41 ± 0.02^a^

Test	Cohesiveness	Adhesiveness (g s)	Elasticity	Chewiness (N)	Hardness (N)
Quercetin	0.58 ± 0.10^a^	−8.09 ± 4.08^a^	0.81 ± 0.03^a^	31.44 ± 3.26^a,b^	67.50 ± 3.21^a^
Vitamin B_2_	0.60 ± 0.01^a^	−16.17 ± 13.83^a^	0.79 ± 0.02^a^	33.07 ± 1.84^a^	69.27 ± 4.64^a^
Controle	0.78 ± 0.05^b^	−2.17 ± 0.09^a^	0.81 ± 0.00^a^	39.86 ± 4.45^b^	72.27 ± 4.86^a^

*Note:* Different superscripts (a, b)differ significantly at *p* ≤ 0.05 according to the Tukey test for each response variable.

As expected, electrical conductivity did not differ significantly among the formulations because all cheeses were produced with the same NaCl/KCl concentration. These results indicate that loading β‐lg nanostructures with quercetin or vitamin B2 did not alter the ionic characteristics of the cheese matrix. Overall, the incorporation of bioactive‐loaded β‐lg nanostructures preserved the physicochemical properties of Minas Frescal cheese, demonstrating the feasibility of developing a bioactive‐enriched formulation without compromising the technological quality of the product.

No significant differences were observed in the mechanical properties of cheeses containing bioactive‐loaded β‐lg nanostructures compared with the control formulation containing unloaded nanostructures, according to Tukey's test. The only exception was cohesiveness, which differed significantly among the treatments. This result suggests that bioactive encapsulation may have slightly modified the interactions between β‐lg nanostructures and the casein matrix, managing to maintain the calcium level in the cheese mass, reducing proteolysis, and maintaining the highest values of cohesiveness.

The absence of significant differences between cheeses containing encapsulated bioactives and the control formulations suggests that the incorporation of nutraceuticals did not disrupt the structural integrity of the β‐lg nanostructures. This behavior can be explained by the molecular interactions governing the encapsulation process. Hydrophobic interactions between the flavonoid quercetin and the β‐lg hydrophobic cavity promote stable binding, while riboflavin may interact with surface residues without inducing significant conformational rearrangements in the protein assembly (Fu et al. 2023; Song et al. 2025).

From a structural point of view, β‐lg nanostructures act as colloidal particles dispersed in the casein network of cheese. In general, precoagulated milk protein particles have an effect similar to that of inert microparticles: they fill intermicellar spaces and can even reinforce the matrix by retaining water and distributing tension (Davidov‐Pardo et al. 2015; Perry and McClements 2020). Gelation studies show that preformed protein aggregates influence coagulation by modifying the dynamics of casein micelles (Panouille et al. 2007; Salvador et al. 2022). In the present case, there was no impairment in network formation, indicating that the β‐lg nanospheres integrated harmoniously into the system. Their presence may have contributed to water retention and electrostatic stabilization of the matrix, without altering the measured hardness or cohesiveness parameters (Table 4).

Therefore, it was possible to add the bioactives in the nanostructure and then add them to the Minas Frescal cheese, without causing significant changes in the physicochemical properties, which is a favorable point for consumer acceptance of this functionally improved product.

## 4 Conclusions

It was possible to produce Minas Frescal cheese with 2.5% total salt (50% NaCl and 50% KCl) and 7.5 mg kg^−1^ of β‐lg nanostructures without altering its physicochemical or textural properties. This formulation is a reduced‐sodium cheese that was obtained by partially replacing NaCl with KCl. It is also enriched with β‐lg nanostructures that encapsulate quercetin or vitamin B2. This demonstrates the feasibility of developing a functional dairy product without compromising its technological quality. Therefore, the technology proposed by this work, based on the results obtained so far, presents a promising potential for the application of the β‐lg nanostructure in the food industry, in terms of improving the texture and functionality of foods. Future studies should include sensory evaluations to determine consumer acceptance, and investigate whether incorporating β‐lg nanostructures and partially replacing NaCl with KCl affects the perception of saltiness, flavor, texture, and the overall acceptability of the cheese.

## Author Contributions

**Brunna D'Onofre Couto:** investigation, data curation, writing – original draft. **Igor José Boggione Santos:** conceptualization, methodology, supervision, writing – review and editing, validation.

## Funding

This work was supported by the Coordenação de Aperfeiçoamento de Pessoal de Nível Superior, Fundação de Amparo à Pesquisa do Estado de Minas Gerais, and Conselho Nacional de Desenvolvimento Científico e Tecnológico.

## Ethics Statement

The authors have nothing to report.

## Conflicts of Interest

The authors declare no conflicts of interest.

## Supporting information


**Figure A.1.** Flowchart of Minas Frescal cheese production with and without β‐lg nanostructures.
**Figure A.2.** Pareto chart for DCCR pH response variable of cheeses with β‐lg nanostructure.
**Figure A.3.** Pareto chart for DCCR Titratable Acidity (%) response variable of cheeses with β‐lg nanostructure.
**Figure A.4.** Pareto chart for DCCR electrical conductivity response variable of cheeses with β‐lg nanostructure.
**Figure A.5.** 2D response surface of the three‐factor DCCR with the dependent variable electrical conductivity as a function of the encoded variables NaCl and KCl concentrations.
**Figure A.6.** 2D response surface of the three‐factor DCCR with the dependent variable electrical conductivity as a function of the encoded variables KCl and β‐lg nanostructures concentrations.
**Figure A.7.** 2D response surface of the three‐factor DCCR with the dependent variable electrical conductivity as a function of the encoded variables NaCl and β‐lg nanostructures concentrations.
**Figure A.8.** Pareto chart for DCCR cohesiviness response variable of cheeses with β‐lg nanostructure.
**Figure A.9.** Pareto chart for DCCR Elasticity response variable of cheeses with β‐lg nanostructure
**Figure A.10.** Pareto chart for DCCR Hardness response variable of cheeses with β‐lg nanostructure.
**Figure A.11.** Pareto chart for DCCR Chewiness response variable of cheeses with β‐lg nanostructure.
**Figure A.12.** Pareto chart for DCCR Adhesiveness response variable of cheeses with β‐lg nanostructure.
**Figure A.13.** 2D response surface of the three‐factor DCCR with the dependent variable adhesiviness as a function of the encoded variables NaCl and KCl concentrations.
**Figure A.14.** 2D response surface of the three‐factor DCCR with the dependent variable adhesiviness as a function of the encoded variables KCl and β‐lg nanostructures concentrations.
**Figure A.15.** 2D response surface of the three‐factor DCCR with the dependent variable adhesiviness as a function of the encoded variables NaCl and β‐lg nanostructures concentrations.
**Table A.1.** Matrix of variable limits: NaCl (g L^−1^), KCl (g L^−1^), and β‐lg nanostructures (mg.kg^−1^).
**Table A.2.** Preparation of different control cheeses (without nanostructure).
**Table A.3.** ANOVA of the electrical conductivity response variable of DCCR of cheeses with β‐lg nanostructures.
**Table A.4.** ANOVA of the Adhesiveness response variable of DCCR of cheeses with β‐lg nanostructures.

## Data Availability

The data that support the findings of this study are available from the corresponding author upon reasonable request.
