# Supplementary material for: Optimization of Salt Composition and Influence of β‐Lactoglobulin Nanostructures on the Physicochemical and Textural Properties of Minas Frescal Cheese
Source: J Texture Stud. 2026 Oct 11;57(5):e70118. doi: 10.1111/jtxs.70118 (PMC13654811; doi:10.1111/jtxs.70118)
Supplement: Supplementary file 1 [file JTXS-57-e70118-s001.docx]

**SUPPLEMENTARY MATERIAL**

**Figure A.1.** Flowchart of Minas Frescal cheese production with and without β-lg nanostructures

Type C

Salt;

Nanostructures;

CaCl_2_

Addition of rennet under stirring

Heating in water bath between 32 and 35°C

Cut

Stirring

Shaping

Draining and turning

Cheese

Storage at 4°C

**Figure A.2**. Pareto chart for DCCR pH response variable of cheeses with β-lg nanostructure

**Figure A.3**. Pareto chart for DCCR Titratable Acidity (%) response variable of cheeses with β-lg nanostructure

**Figure A.4**. Pareto chart for DCCR electrical conductivity response variable of cheeses with β-lg nanostructure

**Figure A.5**. 2D response surface of the three-factor DCCR with the dependent variable electrical conductivity as a function of the encoded variables NaCl and KCl concentrations.

**Figure A.6**. 2D response surface of the three-factor DCCR with the dependent variable electrical conductivity as a function of the encoded variables KCl and β-lg nanostructures concentrations.

**Figure A.7**. 2D response surface of the three-factor DCCR with the dependent variable electrical conductivity as a function of the encoded variables NaCl and β-lg nanostructures concentrations.

**Figure A.8**. Pareto chart for DCCR Cohesiviness response variable of cheeses with β-lg nanostructure

**Figure A.9**. Pareto chart for DCCR Elasticity response variable of cheeses with β-lg nanostructure

**Figure A.10**. Pareto chart for DCCR Hardness response variable of cheeses with β-lg nanostructure

**Figure A.11**. Pareto chart for DCCR Chewiness response variable of cheeses with β-lg nanostructure

**Figure A.12.** Pareto chart for DCCR Adhesiveness response variable of cheeses with β-lg nanostructure

**Figure A.13**. 2D response surface of the three-factor DCCR with the dependent variable Adhesiviness as a function of the encoded variables NaCl and KCl concentrations.

**Figure A.14**. 2D response surface of the three-factor DCCR with the dependent variable Adhesiviness as a function of the encoded variables KCl and β-lg nanostructures concentrations.

**Figure A.15**. 2D response surface of the three-factor DCCR with the dependent variable Adhesiviness as a function of the encoded variables NaCl and β-lg nanostructures concentrations.

**Table A.1.** Matrix of variable limits: NaCl (g.L^-1^), KCl (g.L^-1^) and β-lg nanostructures (mg.kg^-1^)

|  | Variables | -1,67 | -1 | 0 | 1 | 1,67 |
| --- | --- | --- | --- | --- | --- | --- |
| NaCl (g·L^-1^) | | 0.00 | 5.00 | 12.50 | 20.00 | 25.00 |
| KCl (g·L^-1^) | | 25.00 | 20.00 | 12.50 | 5.00 | 0.00 |
| Nano β-lg (mg·kg^-1^) | | 3.32 | 5.00 | 7.50 | 10.00 | 11.68 |

**Table A.2**. Preparation of different control cheeses (without nanostructure)

| Test | Salt Concentrations | | |
| --- | --- | --- | --- |
|  | *NaCl (g·L^-1^)* | *KCl(g·L^-1^)* |  |
| C1 | 5.0 | 20.0 |  |
| C2 | 5.0 | 5.0 |  |
| C3 | 20.0 | 20.0 |  |
| C4 | 20.0 | 5.0 |  |
| C5 | 12.5 | 12.5 |  |
| C6 | 0.0 | 12.5 |  |
| C7 | 25.0 | 12.5 |  |
| C8 | 12.5 | 25.0 |  |
| C9 | 12.5 | 0.0 |  |

**Table A.3.** ANOVA of the electrical conductivity response variable of DCCR of cheeses with β-lg nanostructures

| Factor | SS | Df | MS | F | P |
| --- | --- | --- | --- | --- | --- |
| (1)NaCl (L) | 3,884171 | 1 | 3,884171 | 64,97663 | 0,000087 |
| NaCl (Q) | 0,063235 | 1 | 0,063235 | 1,05782 | 0,337934 |
| (2) KCl (L) | 5,215977 | 1 | 5,215977 | 87,25583 | 0,000033 |
| KCl (Q) | 0,065701 | 1 | 0,065701 | 1,09909 | 0,329309 |
| (3)Nano b-Lg (L) | 0,019915 | 1 | 0,019915 | 0,33314 | 0,581893 |
| Nano b-Lg (Q) | 0,014005 | 1 | 0,014005 | 0,23429 | 0,643138 |
| 1L by 2L | 0,034760 | 1 | 0,034760 | 0,58149 | 0,470641 |
| 1L by 3L | 0,019405 | 1 | 0,019405 | 0,32461 | 0,586661 |
| 2L by 3L | 0,045904 | 1 | 0,045904 | 0,76792 | 0,409924 |
| Error | 0,418446 | 7 | 0,059778 |  |  |
| Total SS | 9,746311 | 16 |  |  |  |

L: Linear; Q: Quadratic

**Table A.4.** ANOVA of the Adhesiveness response variable of DCCR of cheeses with β-lg nanostructures

| Factor | SS | Df | MS | F | P |
| --- | --- | --- | --- | --- | --- |
| (1)NaCl (L) | 395,572 | 1 | 395,5718 | 7,688133 | 0,027585 |
| NaCl (Q) | 292,821 | 1 | 292,8207 | 5,691114 | 0,048481 |
| (2) KCl (L) | 15,981 | 1 | 15,9806 | 0,310591 | 0,594681 |
| KCl (Q) | 1,290 | 1 | 1,2900 | 0,025071 | 0,878661 |
| (3)Nano b-Lg (L) | 0,746 | 1 | 0,7457 | 0,014493 | 0,907558 |
| Nano b-Lg (Q) | 19,158 | 1 | 19,1583 | 0,372351 | 0,561005 |
| 1L by 2L | 5,629 | 1 | 5,6291 | 0,109404 | 0,750504 |
| 1L by 3L | 11,594 | 1 | 11,5940 | 0,225335 | 0,649457 |
| 2L by 3L | 3,661 | 1 | 3,6607 | 0,071147 | 0,797360 |
| Error | 360,166 | 7 | 51,4523 |  |  |
| Total SS | 9,746311 | 16 |  |  |  |

L: Linear; Q: Quadratic
